# Improving Implementation of eMental Health for Mood Disorders in Routine Practice: Systematic Review of Barriers and Facilitating Factors

**DOI:** 10.2196/mental.9769

**Published:** 2018-03-16

**Authors:** Christiaan Vis, Mayke Mol, Annet Kleiboer, Leah Bührmann, Tracy Finch, Jan Smit, Heleen Riper

**Affiliations:** ^1^ Department of Clinical, Neuro-, & Developmental Psychology Faculty of Behavioural and Movement Sciences Vrije Universiteit Amsterdam Amsterdam Netherlands; ^2^ Department of Mental Health Amsterdam Public Health Research Institute Vrije Universiteit Medical Center / Vrije Universiteit Amsterdam Amsterdam Netherlands; ^3^ Department of Research and Innovation Specialized Mental Health Care GGZ InGeest Amsterdam Netherlands; ^4^ Healthcare & Implementation Science Department of Nursing, Midwifery & Health Northumbria University Newcastle upon Tyne United Kingdom; ^5^ Telepsychiatric Unit Faculty of Health Science University Hospital / University of Southern Denmark Odense Denmark

**Keywords:** eMental health, implementation, routine practice, determinants of practices, RE-AIM, barriers and facilitators, mood disorders, review

## Abstract

**Background:**

Electronic mental health interventions (eMental health or eMH) can be used to increase accessibility of mental health services for mood disorders, with indications of comparable clinical outcomes as face-to-face psychotherapy. However, the actual use of eMH in routine mental health care lags behind expectations. Identifying the factors that might promote or inhibit implementation of eMH in routine care may help to overcome this gap between effectiveness studies and routine care.

**Objective:**

This paper reports the results of a systematic review of the scientific literature identifying those determinants of practices relevant to implementing eMH for mood disorders in routine practice.

**Methods:**

A broad search strategy was developed with high sensitivity to four key terms: implementation, mental health care practice, mood disorder, and eMH. The reach, effectiveness, adoption, implementation, and maintenance (RE-AIM) framework was applied to guide the review and structure the results. Thematic analysis was applied to identify the most important determinants that facilitate or hinder implementation of eMH in routine practice.

**Results:**

A total of 13,147 articles were screened, of which 48 studies were included in the review. Most studies addressed aspects of the reach (n=33) of eMH, followed by intervention adoption (n=19), implementation of eMH (n=6), and maintenance (n=4) of eMH in routine care. More than half of the studies investigated the provision of mental health services through videoconferencing technologies (n=26), followed by Internet-based interventions (n=20). The majority (n=44) of the studies were of a descriptive nature. Across all RE-AIM domains, we identified 37 determinants clustered in six main themes: acceptance, appropriateness, engagement, resources, work processes, and leadership. The determinants of practices are expressed at different levels, including patients, mental health staff, organizations, and health care system level. Depending on the context, these determinants hinder or facilitate successful implementation of eMH.

**Conclusions:**

Of the 37 determinants, three were reported most frequently: (1) the acceptance of eMH concerning expectations and preferences of patients and professionals about receiving and providing eMH in routine care, (2) the appropriateness of eMH in addressing patients’ mental health disorders, and (3) the availability, reliability, and interoperability with other existing technologies such as the electronic health records are important factors for mental health care professionals to remain engaged in providing eMH to their patients in routine care. On the basis of the taxonomy of determinants of practices developed in this review, implementation-enhancing interventions can be designed and applied to achieve better implementation outcomes. Suggestions for future research and implementation practice are provided.

## Introduction

### Background

Electronic mental health interventions (eMental health or eMH) for mood disorders such as depression can increase reach and accessibility of mental health services while maintaining comparable clinical outcomes as face-to-face interventions and superior outcomes compared with waiting lists [1-3]. eMH encompasses the use of digital technologies and new media for the delivery of screening, health promotion, prevention, early intervention, treatment, or relapse prevention, as well as for improvement of health care delivery (eg, electronic patient files), professional education (e-learning), and Web-based research in the field of mental health [4]. Research on the translation of the results of these studies into routine care is scarce. Translational research can have two dimensions: dissemination and implementation of an innovation in clinical practice. Dissemination concerns the passive and active spread of information about eMH to relevant stakeholders, including consumers, clinical care providers, and decision- and policy makers. Implementation refers to the process of embedding and integrating new practices into actual care settings [5,6]. It seems that eMH interventions are reasonably well disseminated to clinical practice given that a number of preconditions are fulfilled, such as the availability of technical infrastructures and proper reimbursement of these services [7]. Nevertheless, the actual use of eMH in routine mental health care lags behind expectations. It is unclear why implementation of eMH remains difficult.

A logical approach in addressing this implementation challenge is to identify the factors that might promote or inhibit implementation of eMH in routine practice [8]. On the basis of these determinants, implementation-enhancing interventions might be designed and applied with the aim to improve implementation processes and upscaling of eMH care. Many determinants of different care practices have been identified for a variety of clinical interventions. For example, Krause and colleagues [9] identified over 600 context-specific determinants thought to be relevant in implementing evidence-based interventions for patients with chronic health conditions, including depression in the elderly, chronic obstructive pulmonary disease, and obesity. Examples of these determinants are status and quality of evidence and clinical recommendations, characteristics of the innovation, delivery modalities, reimbursement modalities, implementation leadership, and organizational readiness [10-12]. Similarly, examples of implementation barriers for eMH include the perceived importance of computer literacy skills, knowledge and awareness of existing eMH services, as well as credibility of these services [13]. In turn, many of these determinants have been clustered and framed, currently resulting in more than 60 frameworks used to study and understand implementation processes [14,15]. Although such determinants and frameworks are valuable and comprehensive, they lack specificity to any category of intervention and therefore, provide little practical detail to prioritize determinants and guidance for action to improve the implementation of eMH interventions.

The reach, effectiveness, adoption, implementation, and maintenance (RE-AIM) framework provides a heuristic tool for bridging interventions’ internal validity established in well-controlled conditions and their external validity in real-world conditions [16,17]. It is designed to evaluate the public health impact of health promoting interventions, and it is widely used in implementation research [18]. The framework covers five intervention-related areas of impact: (1) reach as the ability to address those in need of an intervention, (2) effectiveness in terms of the impact of interventions on health outcomes, (3) adoption as a decision to proceed with implementing the clinical intervention, (4) implementation as the process of embedding and integration of the intervention in routine practice and its consistency of delivery and costs, and (5) maintenance as the institutionalization of the intervention in routine care [16,18-20]. Considering the current evidence-base for eMH and the increasing emphasis on comparative effectiveness research in testing clinical and cost-effectiveness of eMH [21], the RE-AIM framework might be a valuable tool to structure determinants of practices that are specific to eMH.

### Research Question

Given the absence of a comprehensive overview of determinants of practices, we systematically reviewed the literature to develop a taxonomy relevant to the implementation of eMH. Knowledge on these determinants can inform the study of interventions that aim to improve the implementation of eMH in routine practice. The following research question guided the research: “What determinants of practice are identified as relevant to implementing eMH interventions for mood disorders in routine practice?” A broad view on eMH and care practice settings, including clinical and community practices, was adopted to provide a comprehensive taxonomy of determinants of mental health practice relevant to implementing eMH.

## Methods

### Study Design

A systematic review of scientific literature was conducted. RE-AIM was used to structure the review. Various implementation studies in the area of mental health care using RE-AIM substantiate the utility of this framework, including evaluations of the implementation of behavior mental health assessment tools [22]; smoking cessation interventions in people with mental illnesses [23]; mental health, substance abuse, and health behavior interventions into specific primary care behavior health programs [24]; tele-mental health consultation program in pediatric primary care in rural settings [25]; and assessing a therapist’s role in eMH for patients with depressive disorders [26].

### Search Strategy

Due to the novelty of the topics concerned (ie, eMH and implementation), a broad search strategy was developed with high sensitivity to four key terms (as opposed to a focused strategy with higher specificity [27]): “implementation,” “mental health care practice,” “mood disorder,” and “eMental-health.” No time frame was applied. On the basis of literature, benchmark definitions for these concepts were developed, and a total of 408 synonyms were formulated for the search strings. A trained librarian guided the formulation of the search strings. The benchmark definitions and search strings are included in Multimedia Appendix 1. The search was conducted in July 2015 in the three main bibliographical databases (PubMed, PsycINFO, and EMBASE). All identified papers were examined for eligibility by two researchers (CV and MM) independently. Disagreements were solved by discussion and, where necessary, moderated by a third researcher (AK) to reach consensus.

### Inclusion and Exclusion Criteria

The inclusion and exclusion criteria are shown in Textboxes 1 and 2.

### Data Extraction

A systematic qualitative narrative approach was applied for the data extraction, analyses, and synthesis of the results [28-30]. A field guide was developed to extract relevant data from the retained articles. Items included the study aim, methods, the psychotherapeutic intervention, eMH technology applied, type of mood disorder, implementation intervention (eg, training of professionals, or a focused marketing campaign to raise awareness of eMH among patients), settings, sample(s), recruitment procedures, results, and findings in terms of determinants of practice. The data were tabulated and categorized in accordance with four of the five RE-AIM dimensions: reach, adoption, implementation, and maintenance. Table 1 presents definitions and adaptations to the RE-AIM dimensions that we applied for the purpose of this study. Effectiveness was not addressed in this review as ample reviews on the clinical effectiveness of eMH for mood disorders are available [1-3]. The implementation dimension was broadened to also include the purposive implementation interventions that might have been employed to achieve better implementation outcomes.

### Analyses and Synthesis

Thematic analysis was applied to identify the recurrent and most important determinants to implementing eMH in routine practice (ie, themes) arising in the included literature. Thematic analysis is a common method for identifying, grouping, and summarizing findings from included studies in narrative review [29]. The (groups of) determinants were developed inductively (ie, without a priori defined topics guiding the analysis). We did not apply a threshold for recurrence of certain themes in the data. Data were extracted by three researchers (CV, MM, and LB) independently. Data files were merged and discrepancies solved by discussion to reach consensus. Freely available reference management software (Mendely, Elsevier), a spreadsheet (Microsoft Excel, Microsoft Corporation), and qualitative analysis software (ATLAS.ti, Scientific Software Development GmbH) were used to organize and conduct the selection, data extraction, and data analysis.

**Inclusion criteria.**

1. Reporting of empirical research such as observational studies using ethnographic methods or experimental studies following a pre-post or randomized controlled trial design
2. The psychotherapeutic intervention under study had an information and communication technology (ICT) component (eg, using videoconferencing, Web, or mobile technologies to deliver mental health care)
3. The psychotherapeutic intervention targeted a mood disorder.
4. The study targeted (1) an adult population, (2) mental health care professionals (HCPs) or, (3) other persons or organizations involved in implementation of eMH.
5. The study took place in routine mental health care settings.

**Exclusion criteria.**

1. Studies were reporting clinical effectiveness data only.
2. The full-text article was not available through Open Access or library loaning services.
3. The full-text article was not available in the English language.

**Table 1:** Dimensions of reach, effectiveness, adoption, implementation, and maintenance (RE-AIM); their definitions; and its focus.

Dimension	Definitions [16]	Comment
Reach	Participation ratio of patients and their characteristics	
Effectiveness	Impact of the (clinical) intervention on patients’ health, quality of life, and economic outcomes	Not addressed in this study
Adoption	Proportion and representativeness of staff and organizations delivering the services	
Implementation	(Clinical) interventions’ fidelity and (implementation) costs	Added: deliberate and purposive actions to implement eMH^a^ [31]
Maintenance	Extent to which the intervention is and remains to be part of routine care practice	

^a^eMH: electronic mental health interventions, or eMental health.

## Results

### Study Selection

**Figure 1:**
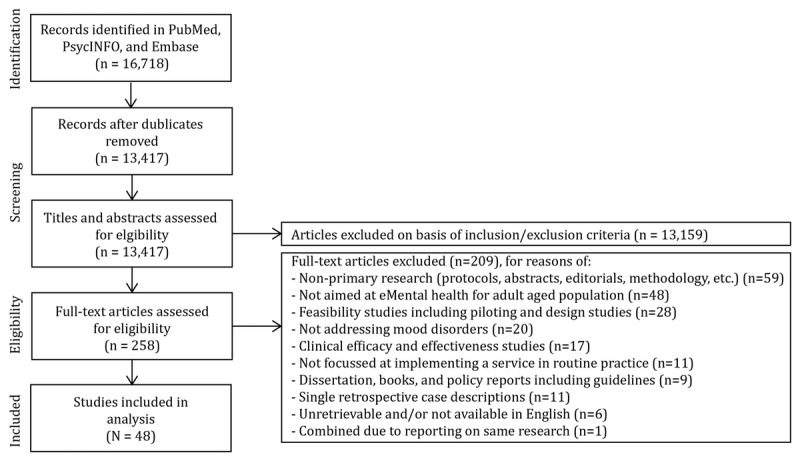
Information flow through the different phases of the systematic review.

The searches resulted in 16,718 records. After removing the duplicates, 13,417 unique titles remained and were screened for eligibility against the inclusion and exclusion criteria. In total, 13,159 articles were excluded on the basis of the information in titles and abstracts. A total of 258 articles were retained, and after examining the full-text articles, a total of 48 studies were included in the analysis. Figure 1 provides an overview of the inclusion and exclusion of studies in the different phases of the systematic review.

### General Study Characteristics

Table 2 provides an overview of the main characteristics of the studies, including the RE-AIM dimension(s) addressed, target disorder, therapeutic principles, technology applied, guidance modalities, and study design.

Most studies investigated reach (n=33), followed by adoption (n=19), implementation (n=6), and maintenance (n=4). The specific type of the target disorder was often described in broad terms such as common mental disorders or mood disorders (n=20), or in exemplary disorders such as depression or anxiety (n=17). Most studies (n=39) did not explicitly report the therapeutic principles of the clinical intervention that was implemented. More than half of the studies investigated the provision of mental health services for mood disorders through videoconferencing technologies (n=26), most often by using videoconferencing for support and consultations. The remainder of the studies focused on Internet-based interventions (n=20). Three studies looked at purely self-help interventions (through Web and mobile technologies), and 10 studies did report on a specific eMH intervention but did not report the guidance modality. Eighteen studies specified the eMH intervention and described the guidance modality. The majority (n=44) of the studies were of an observational, that is, descriptive nature. Most of these (n=20) applied mixed-methods (eg, a survey and semistructured interviews), followed by a large proportion (n=16) of studies that applied qualitative methods such as ethnography or consensus-seeking methods using focus-group discussions. Five studies were of an experimental design, applying either quantitative or mixed-methods. More information about the specific studies’ aims, designs, settings, participants, and clinical and implementation-related interventions are reported in Multimedia Appendix 2.

### Determinants of Practice

**Figure 2:**
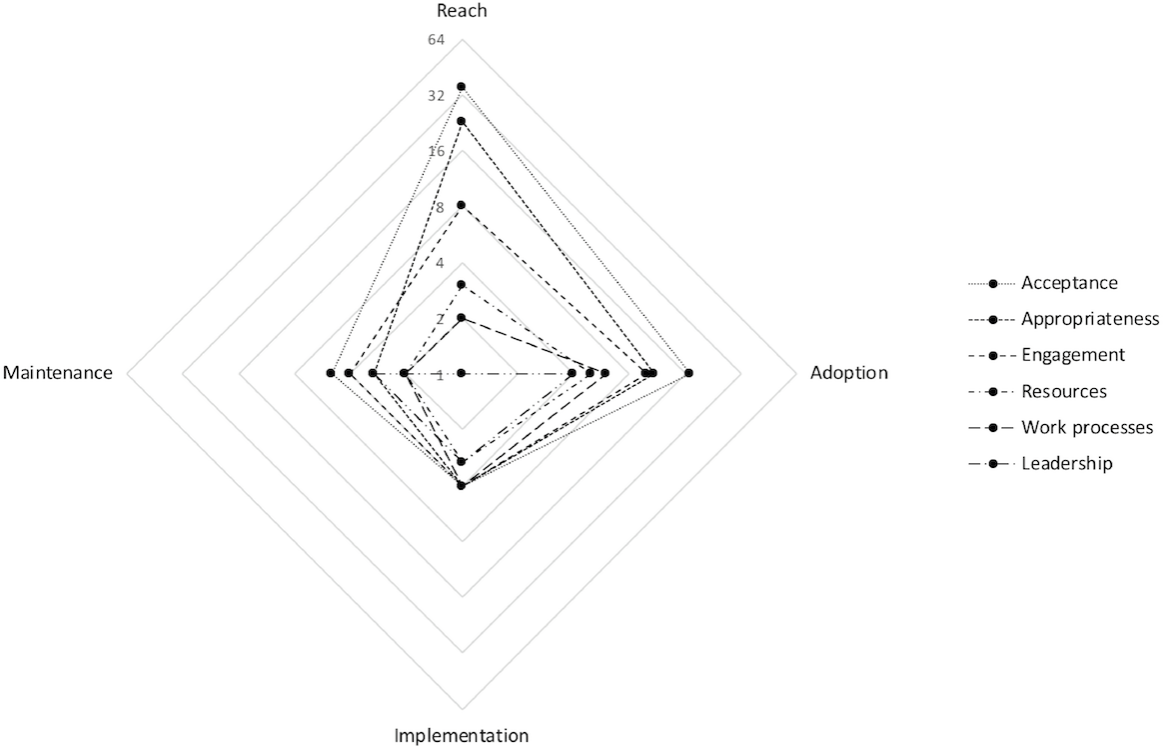
Spider diagram of the spread of the number of studies (n=48) categorized under the RE-AIM dimensions and the six main groups of determinants we identified in literature: acceptance, appropriateness, engagement, resources, work processes, and leadership. RE-AIM: reach, effectiveness, adoption, implementation, and maintenance.

In total, 37 specific determinants of practices relevant to implementing eMH in routine care were identified. The 37 determinants were clustered resulting in a taxonomy of six groups: (1) acceptance of eMH by patients and service delivery staff, (2) appropriateness or clinical relevance of eMH, (3) engagement of participants in implementing and delivering eMH, (4) resources for implementing and delivering eMH, (5) work processes in delivering eMH, and (6) leadership in implementing and delivering eMH. Group definitions are provided in Table 3. The spider diagram in Figure 2 shows that the majority of studies reported determinants in the domain reach that were related to acceptance (n=34) and appropriateness (n=23). When categorized under RE-AIM, reach and the domain adoption were studied most often, addressing determinants related to acceptance (n=17), appropriateness (n=11), and engagement (n=10). Least investigated were the domains of implementation and maintenance.

A detailed list of the determinants is included in Table 4, including their definitions, main perspective, RE-AIM dimensions, and references to the source articles. The following subsections detail the determinants for each of the four RE-AIM domains. The perspective from which become apparent are included, differentiating between (1) patients, (2) staff (individuals and groups) involved in delivering mental health services, (3) organizations as the functional and administrative structures aimed to deliver mental health care, and (4) the system perspective as the human and material resources and organizational arrangements on a community level aimed at to preserve, protect, and restore peoples’ health [32]. More detailed information, including the related excerpts of texts retrieved from the articles, are in Multimedia Appendix 2.

### Reach

The domain reach includes determinants of practices that are related to patients’ participation in eMH and their characteristics. Of the 33 studies that were categorized under reach, most investigated patients’ and mental HCPs perceptions and attitudes of patients and professionals (n=20), or the actual use (n=9) of eMH in a routine care setting. Most studies were of an observational nature (n=31). Two studies used an experimental design for testing interventions aimed at increasing access and use of eMH.

From the perspective of patients, two main groups of factors appeared to be relevant in implementing eMH in routine care: acceptance and appropriateness. Determinants grouped under acceptance concern the perceived and actual feasibility of interacting with eMH. For example, knowledge about the existence of eMH ( awareness, n=13) and technological aspects of the treatment (eg, usability and stability, n=10) were most often reported in the included literature.

Determinants categorized under appropriateness refers to the patients’ perceived fit, relevance, or compatibility of eMH in addressing his or her mental disorder. Within this group, the professional-patient relationship was reported most often by both care providers and patients to be an important aspect that requires consideration when implementing eMH. For example, the perceived importance of interaction and verbal communication was highlighted by van der Vaart, et al [58], showing that the lack in nonverbal communication in Web-based treatments can pose limits to discussing more difficult issues with patients.

From the perspective of staff, engagement emerged as a group of factors next to the determinants grouped under acceptance and appropriateness. Engagement relates to the sustained and effective involvement of staff in implementing and delivering eMH for mood disorders in routine care. Most notably, engagement seem to be related to the organizing structures, policies, and procedures within an organization (n=4), as well as the availability and stability of the required information and communication technology (ICT; n=4). For example, in a qualitative study on expectations of both patients and health professionals in commencing in Internet-based psychotherapy, Montero-Marin et al [48] noted the importance of standardizing Web-based interventions in an integrated service delivery model.

From the perspective of mental health service providing organizations, resources in terms of available and stability of facilitating infrastructure was mentioned (n=2) as an important determinant. In addition, the modus operandi in service delivery both in terms of primary care processes (eg, referral pathways, n=2) as well as facilitating processes (eg, administrative and ICT support and billing processes, n=1) require consideration when implementing eMH in routine practice. Additionally, leadership in terms of existing cultures, strategies, and priorities emerged from the included articles as a determinant of practice (n=1). Regarding the primary care processes, Buist et al [43] showed that considering eMH as a valid service option can influence actual application. Differences in actual use might be caused by differing levels of interest and experience in the eMH service of the service managers.

At health care system level, there were three aspects reported to be of importance, namely policy-making processes (n=2), the availability of appropriate resources including qualified staff (n=2), and collaboration and cooperation within the system and across disciplines (n=1).

**Table 2:** Overview of studies categorized per reach, effectiveness, adoption, implementation, and maintenance (RE-AIM) domain; technology applied; target disorder; therapeutic principles; and study design.

Characteristic		Reach (n=33)	Adoption (n=19)	Implementation (n=6)	Maintenance (n=4)	n^a^
**Target disorder**						
	Depressive disorder	8	3	2	—^b^	10
	Mood disorders^c^	16	9	—	2	20
	Not specified^d^	8	7	4	2	17
**Therapeutic principles**^e^						
	Cognitive behavior therapy	5	3	2	—	8
	Other (eg, mindfulness)	1	—	—	—	1
	General psychotherapy	27	16	4	4	39
**Technology applied**						
	Internet-based (unguided)	2	—	—	—	2
	Internet-based (guided^f^)	3	3	1	—	5
	Internet-based (minimal guidance)	1	—	—	—	1
	Internet-based (therapist guided)	1	—	—	—	1
	Internet-based (blended)	1	1	—	—	1
	Internet-based (not specified^g^)	8	2	1	—	10
	Computer-based	1	1	—	—	1
	mobile health (unguided)	1	—	—	—	1
	Videoconferencing	15	12	4	4	26
**Study design**						
	Experimental—quantitative methods	2	—	—	—	2
	Experimental—mixed-methods	—	2	1	—	3
	Observational—qualitative methods	10	9	2	1	15
	Observational—quantitative methods	6	1	—	1	8
	Observational—mixed-methods	15	7	2	2	20

^a^The n in this column are unique references. Some studies were categorized under more than one RE-AIM dimension.

^b^Refers to no studies categorized under that condition.

^c^Mood disorders including depressive disorder and/or in combination with other mental health disorders.

^d^Refers to the studies that described the target disorder in exemplary wordings without becoming specific. The generic wordings related to mood disorders.

^e^Not all studies specifically discussed the target disorder or psychotherapeutic principles of the service as studies focused, for example, on perceptions of the delivery method relevant to implementation and not on the specific treatment itself.

^f^Some form of guidance; guidance modality and intensity was not specified.

^g^Not specified if it was a guided intervention or self-help.

**Table 3:** Identified groups of determinants of practice and their definitions.

Group	Definition	Determinants
Acceptance	The perception among patients, providers, organizations, and systems that eMH^a^ is agreeable, congenial, or satisfactory.	Access to treatment; expectations and preferences; observability and experience; evidence base; convenience; technology; awareness; skills and competences; privacy; clinical cultures; education; costs; policy; health care system structures
Appropriateness	The perceived fit, relevance, or compatibility of eMH for the patient in addressing his or her mental disorder.	Professional-patient interaction; effectiveness; personal need; flexibility; negative effects; safety; patient characteristics
Engagement	Continuing implementing, delivering, and receiving eMH and remain doing so in the context of concrete treatment plans.	Organizational structures and procedures; leadership; staffing and roles; access and reliability of ICT^b^; time; collaboration
Resources	The availability and appropriateness of resources required in implementing and delivering eMH, including human resources, equipment, funding, and other infrastructural aspects.	Personnel; funds; infrastructure
Work processes	The course of action (modus of operandi) in service delivery and all other tasks and responsibilities mental health care service organizations have.	Primary process; facilitating processes
Leadership	Directing and controlling the working processes and organizing activities that enable implementation and delivery of eMH.	Culture; communication; management; strategies and priorities; external relations

^a^eMH: electronic mental health interventions, or eMental health.

^b^ICT: information and communication technology.

**Table 4:** Determinants of practice identified in the literature mapped on each reach, effectiveness, adoption, implementation, and maintenance (RE-AIM) dimension, including their proposed definitions, main perspective, and references. Indented are determinants grouped within a group of determinants.

Cluster/Determinant		Perspective	RE-AIM^a^	n	References
**Acceptance: the perception among patients and providers that using eMH^b^ is agreeable, congenial, or satisfactory**						
	Access to treatment: the state of accessibility and the act of accessing mental health services.	Patient	R, A	9	[33-41]
	Expectations and preferences: individual and collective attitudes, expectations, and preexisting preferences about receiving and providing mental health care in general and eMH specifically.	Patient	R, A, I	12	[34,37,41-50]
	Expectations and preferences: individual and collective attitudes, expectations, and preexisting preferences about receiving and providing mental health care in general and eMH specifically.	Staff	R, A, I, M	13	[43,48,51-61]
	Observability and experience: the possibility and actual of observations in use (seeing or hearing about the treatment) and experiences of staff in the process of accepting eMH as a valid treatment option.	Staff	R, A, I	7	[43,51-53,59,62,63]
	Evidence-base: the scientific evidence of the feasibility and effectiveness of eMH.	Staff	R, A, I	3	[46,52,61]
	Convenience: the comfort experienced by patients in accessing and receiving mental health care, including overcoming geographical distances, time constraints, and availability of treatment materials.	Patient	R, A, I, M	14	[33,34,39-42,47,59,60,62,64-67]
	Technology: the technical aspects of eMH, including availability of and familiarity with ICT, complexity, usability, and working procedures.	Patient	R, A, M	11	[34,35,37,42,48,49,51,54,55,66,68]
	Convenience: the comfort experienced by patients in accessing and receiving mental health care, including overcoming geographical distances, time constraints, and availability of treatment materials.	Staff	R, A, I, M	8	[43,51-57]
	Technology: the technical aspects of eMH, including availability of and familiarity with ICT, complexity, usability, and working procedures.	Patient	R, A, M	14	[34,37,44-46,48-51,59,60,69-71]
	Technology: the technical aspects of eMH, including availability of and familiarity with ICT, complexity, usability, and working procedures.	Staff	R, A, I, M	8	[43,46,51,53,62,63,71,72]
	Skills and competences: specific personal capacities and means required for receiving (patients) or providing (staff) eMH.	Patient	R, A	7	[33,39,48,51,54,59,73]
	Skills and competences: specific personal capacities and means required for receiving (patients) or providing (staff) eMH.	Staff	R, A, I, M	5	[48,54,55,61,66]
	Privacy: respecting patients’ and providers’ freedom from unauthorized intrusion, including discretion and confidentiality.	Patient	R, A	4	[35,48,49,73]
	Privacy: respecting patients’ and providers’ freedom from unauthorized intrusion, including discretion and confidentiality.	Staff	R, A	1	[48]
	Clinical culture: socially defined and agreed “ways of doing,” including norms, habits, and roles.	Staff	R, A, I, M	6	[43,53,60,61,65,67]
	Education: training of staff in providing eMH in routine care, including technical and therapeutic training, formal education, credentialing, peer-group learning, and supervision.	Staff	R, A, I	13	[43,46,51-53,58,61-63,67,71,72,74]
	Costs: the expenditures made to receive or provide eMH.	Patient	R, A, M	3	[40,66,67]
**Appropriateness: the perceived fit, relevance, or compatibility of eMH for the patient in addressing his or her mental disorder**						
	Professional-patient relationship: the professional interaction between (mental) health care provider and patient, including the aspects such as trust, comfort, and therapeutic interaction.	Patient	R, A, I	18	[33,35,39,40,42,46,48,50,54,55,59, 68-70,73,75-77]
	Professional-patient relationship: the professional interaction between (mental) health care provider and patient, including the aspects such as trust, comfort, and therapeutic interaction.	Staff	R, A, I	10	[46,52,54,55,57-59,61,71,77]
	Effectiveness: patients’ mental health care needs, including information needs and specific (mental) health conditions.	Patient	R	3	[33,35,40]
	Personal need: individual mental health care needs, including information needs and specific (mental) health conditions.	Patients	R, A, M	8	[33,35,42,58,59,65,69,75]
	Flexibility: the extent to which care providers can alter or adapt the eMH to the (perceived) needs of the patient or care provider.	Staff	R, A, I, M	6	[46,58,61,67,69,72]
	Negative effects: the perceived and actual negative (clinical) outcomes of receiving eMH.	Patient	R, A	3	[33,46,78]
	Safety: the physical and mental safety of patients receiving eMH.	Patient	R	3	[35,55,78]
	Safety: the physical and mental safety of patients receiving eMH.	Staff	R, A	3	[52,55,59,69]
	Patient characteristics: individual patient characteristics, including age, gender, clinical history, social economic status, and clinical symptoms relevant to eMH.	Patient	R, A	7	[37,48,69,70,73,78,79]
	Patient characteristics: individual patient characteristics, including age, gender, clinical history, social economic status, and clinical symptoms relevant to eMH.	Staff	R, A, I	4	[43,52,59,61]
**Engagement: continuing implementing, delivering, and receiving eMH and remain doing so in the context of concrete treatment plans**						
	Organizational structures and procedures: the organizing structures, policies, and procedures for delivery of eMH, including standards and clinical guidelines, administrative support, technical support, and other facilitating services.	Staff	R, A, I	8	[43,48,52,55,59,61,62,72]
	Leadership: the managerial capacity and operationalization of an organization, including leadership, goal setting, strategies, and supportive measures	Staff	R, A, I	4	[55,58,62,72]
	Staffing and roles: the availability of staff necessary in delivering eMH, including qualifications, roles, and responsibilities	Staff	R, A, I, M	7	[35,48,53,59,60,62,72]
	Access and reliability of ICT^c^: the availability, stability, and reliability of required technology, including interoperability with other existing technology (eg, electronic patient record).	Staff	R, A, I	10	[43,48,52,53,56,59,62,63,71,72]
	Time: the time constraints in providing mental health care in general and eMH specifically.	Staff	I	1	[61]
	Collaboration: the possibility and actual act of parties involved in delivery of eMH willingly work together, including sharing of information and expertise.	Staff	R, A, I	3	[61,72,77]
**Resources: the availability and appropriateness of resources required in implementing and delivering eMH, including human resources, equipment, funding, and other infrastructural aspects**						
	Personnel: the availability, capacity, and capabilities of persons necessary in the delivering eMH.	Organization	A, I	2	[62,80]
	Funds: the availability and sources of pecuniary resources necessary for delivering eMH and its impact on existing (care) budgets	Organization	A, I, M	3	[66,67,72,80]
	Infrastructure: availability, quality, and stability of facilitating structures required for delivering eMH, including offices and equipment.	Organization	R, A, I, M	7	[43,52,53,60,62,67,72]
**Processes: the course of action (modus of operandi) in service delivery and all other tasks and responsibilities mental health care service organizations have**						
	Primary process: a series of actions conducing to the primary objectives of a mental health care organization such as referral processes, establishing diagnosis, and providing treatment.	Organization	R, A, I, M	7	[43,48,53,60,62,67,80]
	Facilitating processes: the facilitating activities required for primary processes to deliver mental health care services. Facilitating processes do not directly add value to service delivery but are necessary to provide the services.	Organization	R, A, I, M	7	[43,52,60,62,67,72,80]
**Leadership: directing and controlling the working processes and organizing activities that enable implementation and delivery of eMH**						
	Culture: socially defined and agreed “ways of doing,” including norms, habits, and roles relevant to delivering eMH.	Organization	R, A, I, M	2	[43,67]
	Communication: the mechanisms, means, and contents of disseminating information across the mental health care organization.	Organization	A, I	1	[62]
	Management: the managerial capacity and operationalization of an organization delivering eMH, including leadership, goal setting, strategies, and supportive measures.	Organization	A, I, M	3	[60,62,80]
	Strategies and priorities: the operationalization of and operationalized objectives into feasible working plans, including vision, mission, priorities, and work plans.	Organization	R, A, I, M	2	[43,67]
	External relations: cooperation and collaboration of various external parties involved and/or affected by delivery of eMH, including sharing knowledge.	Organization	A, I, M	3	[65,67]
**Health care system: the organization of people, institutions, and resources that deliver mental health care services to meet the health needs of target populations**						
	Policy: the plans or courses of actions intended to influence and determine decisions and actions relevant to delivery of eMH.	Setting	R, A, I, M	2	[43,60]
	Resources: the availability and appropriateness of resources required in delivering eMH, including HCPs^d^, ICT and standardization, funding, and other infrastructural aspects.	Setting	R, M	4	[60,65,70,71]
	Community acceptance: the shared perception among the community that eMH is agreeable, palatable, or satisfactory.	Setting	M	2	[65,66]
	Collaboration: cooperation and collaboration of various parties involved in delivery of eMH, including knowledge sharing.	Setting	R, A, I	1	[43]
	Structure: the organizing and organized plan of health services in a given (geographical) context and relevant to the implementation and delivery of eMH.	Setting	M	1	[60]

^a^RE-AIM: reach, effectiveness, adoption, implementation, and maintenance. Please refer to Table 1 for the specific definitions of the RE-AIM framework. The following abbreviations are used in this column: R: reach; A: adoption; I: implementation; and M: maintenance.

^b^eMH: electronic mental health interventions. or eMental health.

^c^ICT: information and communication technology.

^d^HCPs: health care professionals.

### Adoption

Adoption mirrors the decision of staff and organizations involved in delivering the eMH services and the extent to which they actually use and deploy the services to their patients. Of the 19 studies that were characterized under adoption, 16 studies investigated adoption-related perceptions and attitudes toward eMH (n=9), or actual use (n=7) of eMH in routine care settings showing adoption. Three studies investigated and tested an adoption-enhancing intervention aimed at increasing the number of staff involved in the delivery of eMH.

Seen from the perspective of staff delivering the services, a frequently mentioned determinant grouped under acceptance was patients’ awareness and knowledge of the existence of eMH (n=5). Similarly, the awareness of eMH as a viable treatment option among staff was also identified as a relevant determinant in staff adopting eMH (n=6). Adoption can be facilitated by allowing clinicians to gain experience with eMH and the observability of eMH (n=7). In terms of appropriateness of eMH, the studies indicated that patient-professional relationship is an important determinant to consider when designing interventions aimed at improving adoption rates (n=7). To illustrate, May et al [54] reported on the use of videoconferencing technology in delivering psychotherapy, indicating that the therapist-patient relation should include strategies that appropriately addresses the disorder for which verbal interaction might be essential. Furthermore, the availability and stability of the technical aspects, including infrastructure and interoperability of related ICT (n=8), can be an influential factor in facilitating the engagement of professionals in continuing to offer and apply eMH to their patients.

From the organizations’ perspective, the determinants addressing adoption related mostly to the availability of infrastructural resources (n=5) and the primary care process (n=5). Infrastructural resources included the availability, quality, and stability of facilitating structures such as office rooms and ICT equipment. Determinants related to the primary care processes included issues with referral procedures, diagnostic procedures, and therapy guidelines and manuals. For instance, Jameson et al [53] highlighted that clinical policies and procedures for initiating a referral and coordinating between the various partners involved in service delivery are necessary for successful and sustainable use of eMH.

One article reported determinants from a health care system perspective. Buist et al [43] reported on the importance of mechanisms that enable collaboration, sharing of information, and policies supporting better use of these mechanisms.

### Implementation

Determinants categorized under implementation relate to the extent to which eMH is used in real-world settings as intended (ie, fidelity of use), implementation costs, or deliberate and purposive actions to implement eMH. Of the 6 studies identified under implementation, 2 investigated an implementation-related intervention focusing on training mental health providers to use eMH in daily practice. The other 4 studies performed a process evaluation (n=1) and investigated use and utilization of eMH (n=3).

The most frequently reported determinants from the perspective of staff were related to acceptance. These concerned raising staffs’ awareness about the existence of eMH (n=3) and providing education to staff (n-4) in applying eMH in routine care. Specific determinants included references to technical and therapeutic training, formal education and credentialing, and peer-group learning and supervision. For example, Willhelmsen et al [61] showed the importance of training of general practitioners (GPs) in increasing patients’ acceptance of eMH, which might strengthen the perceived credibility of eMH.

Furthermore, from the perspective of staff, engagement was found to be influenced by the availability of support and facilitating services (n=4). For example, Avey et al [72] reported in a qualitative study on implementation processes that coordination and collaboration between the various persons involved in the service delivery should be facilitated effectively and that a dedicated program coordinator was valued highly among the participating hospitals.

From the viewpoint of an organization, the availability of resources such as staffing (n=2), funding (n=2), and infrastructural facilities (n=2) were reported as relevant determinants. In addition, various factors emerged from the literature related to the primary modes operandi (n=3). For example, Reifels et al [80] discussed that successful implementation might depend on the existence or establishment of effective primary processes in the service delivery structures. Similarly, implementation outcomes can be determined by factors facilitating and supporting the primary processes in delivering mental health care services (n=4). Examples include issues with office space, availability of equipment, and administrative support as Adler et al [62] highlighted. Besides the organizational structures and processes, leadership and management (n=3) need to be considered when implementing eMH. This includes scheduling problems, lack of a clear goals, and managerial support to address issues with existing clinical demands.

From the perspective of health care systems, less rich information was found in the included studies. However, Buist et al [43] did report on determinants of practices relating to the availability of policy measures (n=1) and possibilities to collaborate and share knowledge within and across disciplines and settings (n=1).

### Maintenance

Under maintenance, determinants were categorized that relate to keeping the eMH as a normal part of routine care practices. All 4 maintenance studies were of a descriptive nature aiming to establish usage and utility figures of videoconferencing- delivered mental health services (n=2), capture end-user perceptions (n=1), or describe potential success factors (n=1) of programs that remained in practice after their implementation phase.

From the patients’ viewpoint, the convenience of eMH was seen as an important determinant in maintaining the service in practice (n=4). In an evaluation of patients’ perceptions of a routine tele-psychiatry service in central Alberta, Simpson et al [66] highlighted the importance of reducing waiting times and travel time and that this in the long term might outweigh preferences for face-to-face consultations.

From the perspective of mental health staff, the clinical culture in terms of socially defined and agreed ways of doing (n=2), including norms, habits, and roles, are considered to be important in maintaining the services in routine practice. Hailey et al [65] showed that traditional patterns might keep staff from changing their practice, even if the service is in operation for a considerable time.

At the organizational level, various determinants were reported, including availability of funds (n=2) and infrastructure (n=2), the primary modes of operation (n=2), supporting structures and activities (n=2), and leadership and management (n=3). Regarding the latter, Whitten et al [67] showed in a study comparing tele-psychiatry programs that are in routine care for some time that the different business approaches these programs took might have contributed to their success.

From the perspective of the health care system, besides the importance of policy (n=1), community acceptance (n=2), and organizing and organized plans of health services (ie, structure; n=1), the availability and appropriateness of resources required in maintaining eMH in practice were mentioned (n=2).

## Discussion

### Principal Findings

We developed a taxonomy of 37 determinants of mental health care practices known in the literature as relevant to successfully implement eMH for mood disorders. The determinants of practices clustered in six groups are expressed at (a combination of) patient, staff, organization, and setting levels and address one or more RE-AIM dimensions (see Table 3). Three determinants were reported most frequently: (1) acceptance of eMH in terms of the expectations and preferences of patients and professionals; (2) appropriateness of eMH in addressing the mental health disorder, and specifically, the therapeutic interactions mediated by eMH; and (3) the availability, stability, and reliability of required technologies, including successful interoperability with other existing technologies.

### Strengths and Limitations

The search strategy in this review aimed to capture as much relevant scientific literature as possible. For this reason, broadly defined search terms were used. By applying a standardized integrative approach (RE-AIM in combination with qualitative thematic analysis), we were able to search for commonalities in the concepts and underlying study characteristics while preserving the heterogeneous nature of the data retrieved from the studies. However, and although we searched three important bibliographic databases, it is likely that important work from social scientist generalist databases was excluded.

The evidence supporting the determinants identified in this study is mostly of a descriptive nature obtained from observational studies. Due to the limited empirical evidence verifying causality of specific determinants of practices and implementation successes, the findings of this work should be interpreted with care. In an attempt to substantiate this, we conducted a quality appraisal analysis. We included a wide variety of studies ranging from observational case studies using qualitative ethnographic methods to randomized controlled trials quantitatively testing specific implementation interventions. However, because of the heterogeneity of these studies and the absence of validated instruments to assess quality, it proved impossible to come to sensible conclusions about the quality of the evidence. An elaborate approach as done by Greenhalgh et al [81,82], meta-narrative approach in developing a model of diffusion of innovations by including the research traditions from which the included studies emerged might be a fruitful approach but was beyond the scope of this review.

### Comparison With Other Work

Drozd et al [83] conducted a scoping review of 164 publications (including gray literature). The investigators applied the Active Implementation Framework (AIF) to identify implementation-related factors [84]. The AIF describes the components of an implementation practice, including aspects of staff and patient selection, training, supervision, performance assessment, decision support, administrative support, system intervention, and leadership. Drozd and colleagues found in their review factors similar to those that emerged from our analysis of the literature, including certain competences of patients and professionals and organizational drivers. Regarding the latter, the authors did not find empirical support for determinants such as leadership. The authors conclude that not finding empirical evidence for organizational drivers merely indicates a gap in the implementation-related research. Despite the low numbers (n=4), our study shows that leadership indeed is found in empirical research to be a relevant determinant in implementing eMH. This difference can perhaps be explained by the methodological choices that were made for reviewing the literature. Where Drozd and colleagues choose to follow a top-down approach (the AIF), our review followed a quantitative inductive process in identifying the topics related to implementing eMH that emerged from the included articles. Furthermore, the search strategy and data sources in light of their quality and comparability most likely influenced the results.

Similarly, Ross et al [85] updated a systematic review (of reviews, n=44) and looked at qualitative accounts of factors that influence implementation of eHealth interventions in a broader context, including somatic care. Factors identified by these researchers are comparable with the ones presented here, including complexity factors and adaptability, adding to the users’ perception of the acceptability of eHealth interventions. However, it should be noted that the concept of eHealth used by the authors included a variety of ICT-mediated health care services in four main categories: management systems, communication systems, clinical decision support systems, and information systems. In this respect, the authors did not address eHealth to contain purposed intrinsic therapeutic content aimed at improving health conditions as we did. This raises the question of whether generic eHealth both in terms of care setting (health care in general vs mental health care for mood disorders) and purpose (information sharing, support systems vs therapeutic interventions focusing on care and cure) give rise to (partial) different taxonomies of determinants of practice.

### Recommendations for Implementation Practice

Implementation practitioners might benefit in implementing eMH in routine care practices by taking into account the barriers and facilitators that are identified in this systematic review. Specific implementation activities can be designed and applied on the basis of these factors to achieve better implementation outcomes.

One of the most frequently mentioned barriers emerging from the literature concerns the expectations and preferences of patients and professionals about eMH services. Negative individual and collective attitudes, expectations, and existing preferences can prohibit successful implementation of eMH. Ebert et al [45] showed that providing information to patients can enhance their acceptance of eMH. In addressing expectations and preferences of mental health care staff, it is advisable to include service delivery staff in the early stages of decision making and strategy development to increase acceptance and inform concrete implementation activities aimed at the concerns of the end users.

A second important determinant of practice is related to the appropriateness of the eMH intervention in addressing the mental disorder. Within this cluster, the nature and quality of the interactions between the professional and the patient is thought to be highly influential in obtaining favorable clinical outcomes. This includes aspects such as building trust, comfort, and the quality of the therapeutic interactions. eMH interventions delivered through ICT are thought to influence these interactions negatively. Hadjistavropoulos et al [74,86] showed that specific training can change knowledge about, attitudes toward and confidence in delivering eMH. Careful development of training programs and (continuous) guidance of HCPs in applying the eMH intervention might lower barriers with perceived patient-professional interaction through eMH. In addition, innovative models of for integrating therapist support in eMH services might address issues with engagement and the patient-professional relationship [87].

Third, the availability and reliability of required technologies is considered an important determinant for mental HCPs to remain engaged in providing eMH to their patients in routine care. This includes the interoperability with other existing technologies such as electronic health records. It seems important to ensure that the user perspective, including that of the service delivery staff, is taken into account and that the eMH service seamlessly fits within existing technologies and work processes. Here, single-sign on technology and intelligent portal designs might be fruitful avenues to explore.

### Future Research

To increase impact and added value of future research on implementation of eMH for mood disorders in routine practice, the following two topics should be taken into account: (1) identifying organization and system-level determinants and (2) empirical evidence on the effects of implementation strategies in addressing specific barriers and exploiting facilitating factors.

Until now, most implementation research was focused on practitioner and patient-level determinants. Service delivery takes place in a social context at micro (individuals, teams), mesa (organizations), and macro (systems) level. Knowledge about how these different contexts influence implementation efforts can facilitate further scaling up of eMH. Research on systems level might focus on the possible policy measures that enhance implementation of eMH at service deliverer level. For example, what resources at organization or health care system-level are required to deliver eMH? This can include processes of task shifting, curricula and certification of mental health staff, ICT and standardization, funding, and other infrastructural aspects. Or, what role does community acceptance have in implementing eMH in routine practice, and how can the shared perception of community as a whole be changed? Detailed knowledge of organization and setting level factors might be more likely to come from a combination of clinical psychology, social sciences, organizational psychology, and policy research. Here, the MasterMind project [88] might provide inspiration for further research on determinants of practices of eMH.

Furthermore, the field would benefit from well-performed experiments designed to test implementation interventions addressing specific determinants of practices. As shown in this review, there is limited evidence on the causal relationship between determinants and implementation outcomes. Well-designed experiments studying the effects of to the local context–tailored implementation strategies might contribute to the understanding of mechanisms of implementation processes. Do, for example, educational meetings (and in what formats) contribute in raising awareness among GPs about which patient might benefit most from which eMH intervention? Or can championing an Internet-based cognitive behavioral therapy service increase the adoption of other therapists in mental health care team while maintaining the flexibility therapists need to adapt parts of the treatments to the patients’ needs? Fusing implementation practices and research into natural implementation laboratories might be a valuable approach to engage in comparative effectiveness studies of implementation interventions. In these types of studies, experimental implementation interventions can be compared with usual implementation activities for their effects on the degree of normalization of a clinical intervention in real-world service delivery settings. The ImpleMentAll project (project position paper and study protocol forthcoming) might be a good example of this approach. This type of future research might lead to a shift from practice-based and evidence-informed to evidence-based implementation of clinically effective and relevant eMH interventions.

### Conclusions

This study systematically reviewed scientific literature and developed an evidence-informed taxonomy of six clusters of 37 determinants of practices we found in literature: (1) acceptance of eMH interventions among patients, providers, organizations, and health care settings; (2) appropriateness of eMH interventions in addressing the disorder; (3) engagement in implementing, delivering, and receiving eMH interventions and remain doing so; (4) the availability and appropriateness of resources for implementing and delivering eMH interventions; (5) processes relating to the modus of operandi in delivering eMH interventions; and (6) leadership directing and controlling processes and organizing activities enabling implementation and delivery of eMH interventions. On the basis of these determinants of practices, implementation-enhancing interventions can be designed, tested, and applied to achieve better implementation outcomes. Suggestions for implementation practice are discussed, such as in-depth training of professionals, careful selection, and continuous development of the eMH technology used. In addition, focal points for future research are provided, including implementation-related factors on organization and system level, as well as (quasi) experimental research to test the effectiveness of specific implementation interventions in attaining better implementation outcomes for eMH service provision.

## Appendix

Multimedia Appendix 1 Benchmark definitions, definitions of RE-AIM, and the actual search strings that were applied in the search and analysis strategy.

Multimedia Appendix 2 Data file with two sheets. Sheet 1 contains the aims, designs, settings, participants, and clinical and implementation-related interventions of the studies that were included in this review. Sheet 2 lists the determinants and supporting excerpts of texts retrieved from the articles that were included in the thematic analysis.

## Abbreviations

AIF: Active Implementation Framework
eMH: electronic mental health interventions or eMental health
GP: general practitioner
HCPs: health care professionals
ICT: information and communication technology
RE-AIM: reach, effectiveness, adoption, implementation, and maintenance
