# Supplementary material for: Improving Implementation of eMental Health for Mood Disorders in Routine Practice: Systematic Review of Barriers and Facilitating Factors
Source: JMIR Ment Health. 2018 Mar 16;5(1):e20. doi: 10.2196/mental.9769 (PMC5878369; doi:10.2196/mental.9769)
Supplement: Multimedia Appendix 1 [file mental_v5i1e20_app1.pdf]

Improving implementation of eMental-health for mood disorders in routine practice: a systematic review of barriers and facilitating factors

Vis C, Mol M, Kleiboer A, Bührmann L, Finch T, Smit J, Riper H

Appendix 1 – Benchmark definitions, RE-AIM, and search strings

Inclusion criteria

- Intervention / program: eMental health and innovations targeting at common mental disorders (benchmark definitions)
- Participants: adults; patients; professionals; mental healthcare organisations
- Settings: Routine mental healthcare practice (benchmark definition)
- Outcomes: Implementation relevant outcomes in terms of Reach, Adoption, Implementation and Maintenance (RE-AIM definitions)

Exclusion criteria

- Clinical effectiveness and cost-effectiveness as primary outcomes (E in RE-AIM)
- Not in English language
- Non-primary research (e.g. protocols, books, literature reviews, editorials, letters commentaries, opinions, addendums, etc.)

Benchmark definitions

Implementation: "the active and planned efforts to mainstream an innovation within an organization" [1]. In terms of institutionalization or normalization, mainstreaming an innovation can be understood as the set of activities to intentionally embed and integrate practices into their (social) contexts" [2]. Implementation on individual level includes the "decision to make full use of the innovation as the best course of action available" [3], and on organisational level, the means to adapt or assimilate the innovation to the environment in which it is implemented [4]. Assimilation in that sense includes the decision-making and the process that might lead to "the innovation's full acceptance, utilisation and institutionalization" [5].

Innovation: "An innovation is an idea, practice or object that is perceived as new by an individual or other unit of adoption." [3]. In line with Greenhalgh et al.'s deliberation about the concept of innovation in health services [6], the idea, practice or object include behaviours, routines and ways of working, and associated technologies and systems. The new practice should be aimed at improving health outcomes, efficiency, cost-effectiveness, or experience. The object regarded as 'new' is interpreted in relation to previous practices, and should be determined so by a proportion of key stakeholders involved in that practice.

Mental healthcare routine practice: organized healthcare by which persons receive services for mental health problems. These services can differ from screening, diagnose, treatment to relapse prevention. Focus on common mental health problems: depression and anxiety?

eMental health: "E-mental health encompasses the use of digital technologies and new media for the delivery of screening, health promotion, prevention, early intervention, treatment, or

relapse prevention as well as for improvement of health care delivery (e.g. electronic patient files), professional education (e-learning), and online research in the field of mental health." [7].

Definitions of RE-AIM

REACH (patient level). Reach refers to the percentage and risk characteristics of persons (clients, patients) who receive or are affected by a policy or program. It includes both a ratio of participation and characteristics of participants to understand "the degree to which a program reaches those in need" [8]. Reach is also operationalized in terms of the program's ability to "attract its intended audience" [9].

EFFECTIVENESS (patient level). Defined as the impact of an intervention on outcomes, including potential negative effects, quality of life, and economic outcomes [10]. This dimension is not addressed in current review

ADOPTION (setting and therapist level). Adoption refers to the proportion and representativeness of settings (e.g. clinics, work sites etc.) or staff (therapists etc) to adopt a given policy or program [8]. It is defined as "the absolute number, proportion, and representativeness of settings (e.g., health departments) and interventionists (e.g., nurses, educators) who deliver a program" [11].

IMPLEMENTATION (setting level). Implementation is also referred to as "intervention fidelity" to the various elements of an intervention' [9] and is defined in literature as "intervention agents' fidelity to the intervention's protocol" and includes consistency of delivery and cost of the intervention [10].

MAINTENANCE (setting level). Maintenance (at the setting level) is the extent to which a program or policy "becomes institutionalized or part of the routine organizational practices and policies" [10]. In that sense it also is referred to as the sustainability of an intervention in routine practice.

Search strings

Pubmed

"Health Plan implementation"[Mesh] OR "Diffusion of Innovation"[Mesh] OR "Program Evaluation"[Mesh] OR "Nursing Evaluation Research"[Mesh] OR "Health care reform"[Mesh] OR "Delivery of Health Care"[Mesh] OR "Organizational Innovation"[Mesh] OR "Information Dissemination"[Mesh] OR "Translational Medical Research"[Mesh] OR implement[tiab] OR implementation[tiab] OR implemented[tiab] OR implementing[tiab] OR diffuse[tiab] OR diffused[tiab] OR diffusion[tiab] OR disseminate[tiab] OR disseminated[tiab] OR disseminating[tiab] OR dissemination[tiab] OR upscale[tiab] OR up-scale[tiab] OR uptake[tiab] OR up-take[tiab] OR translation[tiab] OR translational[tiab] OR program evaluation[tiab] OR process evaluation[tiab] OR normalisation[tiab] OR normalise[tiab] OR normalised[tiab] OR normalising[tiab] OR normalization[tiab] OR normalize[tiab] OR normalized[tiab] OR normalizing[tiab] OR mainstream[tiab] OR mainstreamed[tiab] OR mainstreaming[tiab] OR maintainance[tiab] OR maintained[tiab] OR sustain[tiab] OR sustainability[tiab] OR sustainable[tiab] OR sustained[tiab] OR integrate[tiab] OR integrated[tiab] OR integrating[tiab] OR integration[tiab] OR nursing evaluation research [tiab] OR health care reform [tiab] OR delivery of health care [tiab] OR delivery of healthcare [tiab] OR organizational innovation [tiab] OR implement[ot] OR implementation[ot] OR implemented[ot] OR implementing[ot] OR diffuse[ot] OR diffused[ot] OR diffusion[ot] OR disseminate[ot] OR disseminated[ot] OR disseminating[ot] OR dissemination[ot] OR

upscale[ot] OR uptake[ot] OR up-take[ot] OR translation[ot] OR translational[ot] OR program evaluation[ot] OR process evaluation[ot] OR normalisation[ot] OR normalised[ot] OR normalization[ot] OR normalize[ot] OR normalized[ot] OR normalizing[ot] OR mainstream[ot] OR mainstreaming[ot] OR maintainance[ot] OR maintained[ot] OR sustain[ot] OR sustainability[ot] OR sustainable[ot] OR sustained[ot] OR integrate[ot] OR integrated[ot] OR integrating[ot] OR integration[ot] OR nursing evaluation research [OT] OR health care reform [OT] OR delivery of health care [OT] OR delivery of healthcare [OT] OR organizational innovation [OT]

AND

"Mental Health Services"[Mesh] OR "Inpatients"[Mesh] OR "Outpatients"[Mesh] OR "Community Psychiatry"[Mesh] OR "Hospitalization"[Mesh] OR mental health service[tiab] OR mental health services[tiab] OR mental healthcare[tiab] OR mental health care[tiab] OR mental practice[tiab] OR mental practices[tiab] OR mental service delivery[tiab] OR inpatient care[tiab] OR in-patient care[tiab] OR out-patient care[tiab] OR outpatient care[tiab] OR mental health program[tiab] OR mental health programme[tiab] OR mental health programmes[tiab] OR mental health programs[tiab] OR community psychiatry[tiab] OR community psychology[tiab] OR community based[tiab] OR mental health service[ot] OR mental health services[ot] OR mental healthcare[ot] OR mental health care[ot] OR mental practice[ot] OR inpatient care[ot] OR out-patient care[ot] OR outpatient care[ot] OR mental health program[ot] OR mental health programme[ot] OR mental health programs[ot] OR community psychiatry[ot] OR community psychology[ot] OR community based[ot] OR "Depression"[Mesh] OR "Depressive Disorder"[Mesh] OR "Mood Disorders"[Mesh] OR "Bipolar Disorder"[Mesh] OR mood disorder[tiab] OR mood disorders[tiab] OR depressed[tiab] OR depressing[tiab] OR depression[tiab] OR depressions[tiab] OR depressive[tiab] OR bipolar[tiab] OR mania[tiab] OR manic[tiab] OR mood disorder[ot] OR mood disorders[ot] OR depressed[ot] OR depressing[ot] OR depression[ot] OR depressions[ot] OR depressive[ot] OR bipolar[ot] OR mania[ot] OR manic[ot]

AND

"Telecommunications"[Mesh] OR "Computer Systems"[Mesh] OR "Mobile Applications"[Mesh] OR telehealth[tiab] OR tele-health[tiab] OR telemedicine[tiab] OR tele-medicine[tiab] OR e-health[tiab] OR ehealth[tiab] OR emental[tiab] OR e-mental[tiab] OR mhealth[tiab] OR m-health[tiab] OR web-based intervention[tiab] OR web-based interventions[tiab] OR web-based treatment[tiab] OR web-based treatments[tiab] OR web-based therapy[tiab] OR web-based therapies[tiab] OR internet intervention[tiab] OR internet interventions[tiab] OR internet treatment[tiab] OR internet treatments[tiab] OR internet therapy[tiab] OR internet therapies[tiab] OR internet-based intervention[tiab] OR internet-based interventions[tiab] OR internet-based treatment[tiab] OR internet-based treatments[tiab] OR internet-based therapy[tiab] OR internet-based therapies[tiab] OR online intervention[tiab] OR online interventions[tiab] OR online treatment[tiab] OR online treatments[tiab] OR online therapy[tiab] OR online therapies[tiab] OR computer-based intervention[tiab] OR computer-based interventions[tiab] OR computer-based treatment[tiab] OR computer-based treatments[tiab] OR computer-based therapy[tiab] OR phone-based intervention[tiab] OR phone-based interventions[tiab] OR phone-based treatment[tiab] OR telephone-based intervention[tiab] OR telephone-based interventions[tiab] OR telephone-based treatment[tiab] OR smartphone-based intervention[tiab] OR smartphone-based interventions[tiab] OR guided[tiab] OR unguided[tiab] OR blended[tiab] OR computer mediated[tiab] OR computer assisted[tiab] OR computer augmented[tiab] OR econsult[tiab] OR econsultation[tiab] OR econsultations[tiab] OR e-consult[tiab] OR e-consultation[tiab] OR e-consultations[tiab] OR remote consultation[tiab] OR remote consultations[tiab] OR teleconsult[tiab] OR

teleconsultation[tiab] OR teleconsultations[tiab] OR tele-consult[tiab] OR tele-consultation[tiab] OR tele-consultations[tiab] OR telemonitoring[tiab] OR tele-monitoring[tiab] OR electronic communication[tiab] OR electronic communications[tiab] OR electronic mail[tiab] OR email[tiab] OR e-mail[tiab] OR emailing[tiab] OR e-mailing[tiab] OR telecommunication[tiab] OR telecommunications[tiab] OR tele-communication[tiab] OR tele-communications[tiab] OR teleconference[tiab] OR teleconferences[tiab] OR teleconferencing[tiab] OR tele-conference[tiab] OR tele-conferences[tiab] OR tele-conferencing[tiab] OR videoconference[tiab] OR videoconferences[tiab] OR videoconferencing[tiab] OR video-conference[tiab] OR video-conferences[tiab] OR telehealth[ot] OR tele-health[ot] OR telemedicine[ot] OR tele-medicine[ot] OR e-health[ot] OR ehealth[ot] OR emental[ot] OR e-mental[ot] OR mhealth[ot] OR m-health[ot] OR web-based intervention[ot] OR web-based interventions[ot] OR web-based treatment[ot] OR web-based treatments[ot] OR web-based therapy[ot] OR web-based therapies[ot] OR internet intervention[ot] OR internet interventions[ot] OR internet treatment[ot] OR internet therapy[ot] OR internet therapies[ot] OR internet-based intervention[ot] OR internet-based interventions[ot] OR internet-based treatment[ot] OR internet-based treatments[ot] OR internet-based therapy[ot] OR online intervention[ot] OR online interventions[ot] OR online treatment[ot] OR online therapy[ot] OR computer-based intervention[ot] OR computer-based interventions[ot] OR computer-based therapy[ot] OR telephone-based intervention[ot] OR guided[ot] OR unguided[ot] OR blended[ot] OR computer mediated[ot] OR computer assisted[ot] OR computer augmented[ot] OR econsultation[ot] OR e-consultation[ot] OR remote consultation[ot] OR teleconsultation[ot] OR tele-consultation[ot] OR telemonitoring[ot] OR tele-monitoring[ot] OR electronic communication[ot] OR electronic mail[ot] OR email[ot] OR e-mail[ot] OR telecommunication[ot] OR telecommunications[ot] OR teleconference[ot] OR teleconferences[ot] OR teleconferencing[ot] OR videoconference[ot] OR videoconferencing[ot] OR video-conference[ot]

Psychinfo

DE "Mental Health Program Evaluation" OR DE "Program Development" OR DE "Program Evaluation" OR "Innovation" OR DE "Health Maintenance Organization" OR DE "Utilization Reviews" OR DE "Health Care Delivery" OR DE "Health Care Utilization" OR DE "Information Dissemination" OR DE "Mainstreaming" OR DE “Health Care Reform” OR TI implement OR TI implementation OR TI implemented OR TI implementing OR TI diffuse OR TI diffusion OR TI diffused OR TI disseminate OR TI disseminated OR TI disseminating OR TI dissemination OR TI upscale OR TI up-scale OR TI uptake OR TI up-take OR TI translational OR TI translation OR TI program evaluation OR TI process evaluation OR TI program development OR TI normalization OR TI normalisation OR TI normalized OR TI normalised OR TI normalise OR TI normalize OR TI normalising OR TI normalizing OR TI mainstream OR TI mainstreamed OR TI mainstreaming OR TI maintained OR TI maintenance OR TI sustain OR TI sustainable OR TI sustained OR TI sustainability OR TI integration OR TI integrated OR TI integrate OR TI integrating OR TI ‘organizational innovation’ OR TI ‘utilization reviews’ OR TI delivery of healthcare OR TI delivery of health care OR AB implement OR AB implementation OR AB implemented OR AB implementing OR AB diffuse OR AB diffusion OR AB diffused OR AB disseminate OR AB disseminated OR AB disseminating OR AB dissemination OR AB upscale OR AB up-scale OR AB uptake OR AB up-take OR AB translational OR AB translation OR AB program evaluation OR AB process evaluation OR AB program development OR AB normalization OR AB normalisation OR AB normalized OR AB normalised OR AB normalise OR AB normalize OR AB normalising OR AB normalizing OR AB mainstream OR AB mainstreamed OR AB mainstreaming OR AB maintained OR AB maintenance OR AB sustain OR AB sustainable OR AB sustained OR AB sustainability OR AB integration OR AB integrated

OR AB integrate OR AB integrating OR AB ‘organizational innovation’ OR AB ‘utilization reviews’ OR AB ‘delivery of healthcare’ OR AB ‘delivery of health care’

AND

DE "Community Mental Health Services" OR DE "Mental Health Services" OR DE "Community Mental Health" OR DE "Community Mental Health Centers" OR DE "Community Psychiatry" OR DE "Psychiatric Units" OR DE "Partial Hospitalization" OR DE "Hospitalization" OR DE "Psychiatric Hospitalization" OR TI mental health service OR TI mental health services OR TI mental healthcare OR TI mental health care OR TI mental practice OR TI mental service delivery OR TI mental health center OR TI mental health centers OR TI inpatient care OR TI in-patient care OR TI out-patient care OR TI outpatient care OR TI mental health program OR TI mental health programs OR TI community psychiatry OR TI community psychology OR TI community based OR AB mental health service OR AB mental health services OR AB mental healthcare OR AB mental health care OR AB mental practice OR AB mental service delivery OR AB mental health center OR AB mental health centers OR AB inpatient care OR AB in-patient care OR AB out-patient care OR AB outpatient care OR AB mental health program OR AB mental health programs OR AB community psychiatry OR AB community psychology OR AB community based OR DE "Anaclitic Depression" OR DE "Dysthymic Disorder" OR DE "Endogenous Depression" OR DE "Postpartum Depression" OR DE "Reactive Depression" OR DE "Recurrent Depression" OR DE "Treatment Resistant Depression" OR DE "Depression (Emotion)" OR DE "Bipolar Disorder" OR DE "Major Depression" OR DE "Mania" OR DE “Affective disorders” OR TI mood disorder OR TI mood disorders OR TI affective disorder OR TI affective disorders OR TI depression OR TI depressed OR TI depressive OR TI bipolar OR TI mania OR TI manic OR AB mood disorder OR AB mood disorders OR AB affective disorder OR AB affective disorders OR AB depression OR AB depressed OR AB depressive OR AB bipolar OR AB mania OR AB manic

AND

DE "Telemedicine" OR DE "Internet" OR DE "Electronic Communication" OR DE "Teleconferencing" OR DE "Mobile Devices" OR DE "Cellular Phones" OR TI telehealth OR TI tele-health OR TI telemedicine OR TI tele-medicine OR TI e-health OR TI ehealth OR TI emental OR TI e-mental OR TI mhealth OR TI m-health OR TI web-based intervention OR TI web-based interventions OR TI web-based treatment OR TI web-based treatments OR TI web-based therapy OR TI web-based therapies OR TI internet intervention OR TI internet interventions OR TI internet treatment OR TI internet treatments OR TI internet therapy OR TI internet therapies OR TI internet-based intervention OR TI internet-based interventions OR TI internet-based treatment OR TI internet-based treatments OR TI internet-based therapy OR TI internet-based therapies OR TI online intervention OR TI online interventions OR TI online treatment OR TI online treatments OR TI online therapy OR TI online therapies OR TI computer-based intervention OR TI computer-based interventions OR TI computer-based treatment OR TI computer-based treatments OR TI computer-based therapy OR TI phone-based intervention OR TI phone-based interventions OR TI phone-based treatment OR TI telephone-based intervention OR TI telephone-based interventions OR TI telephone-based treatment OR TI smartphone-based intervention OR TI smartphone-based interventions OR TI guided OR TI unguided OR TI blended OR TI computer mediated OR TI computer assisted OR TI computer augmented OR TI econsult OR TI econsultation OR TI econsultations OR TI e-consult OR TI e-consultation OR TI e-consultations OR TI remote consultation OR TI remote consultations OR TI teleconsult OR TI teleconsultation OR TI teleconsultations OR TI tele-consult OR TI tele-consultation OR TI tele-consultations OR TI telemonitoring OR TI tele-monitoring OR TI electronic communication OR TI electronic communications OR TI electronic mail OR TI email OR TI e-mail OR TI emailing OR TI e-mailing OR TI telecommunication OR TI telecommunications OR TI tele-communication OR TI tele-

communications OR TI teleconference OR TI teleconferences OR TI teleconferencing OR TI tele-conference OR TI tele-conferences OR TI tele-conferencing OR TI videoconference OR TI videoconferences OR TI videoconferencing OR TI video-conference OR TI video-conferences OR AB telehealth OR AB tele-health OR AB telemedicine OR AB tele-medicine OR AB e-health OR AB ehealth OR AB emental OR AB e-mental OR AB mhealth OR AB m-health OR AB web-based intervention OR AB web-based interventions OR AB web-based treatment OR AB web-based treatments OR AB web-based therapy OR AB web-based therapies OR AB internet intervention OR AB internet interventions OR AB internet treatment OR AB internet treatments OR AB internet therapy OR AB internet therapies OR AB internet-based intervention OR AB internet-based interventions OR AB internet-based treatment OR AB internet-based treatments OR AB internet-based therapy OR AB internet-based therapies OR AB online intervention OR AB online interventions OR AB online treatment OR AB online treatments OR AB online therapy OR AB online therapies OR AB computer-based intervention OR AB computer-based interventions OR AB computer-based treatment OR AB computer-based treatments OR AB computer-based therapy OR AB phone-based intervention OR AB phone-based interventions OR AB phone-based treatment OR AB telephone-based intervention OR AB telephone-based interventions OR AB telephone-based treatment OR AB smartphone-based intervention OR AB smartphone-based interventions OR AB guided OR AB unguided OR AB blended OR AB computer mediated OR AB computer assisted OR AB computer augmented OR AB econsult OR AB econsultation OR AB econsultations OR AB e-consult OR AB e-consultation OR AB e-consultations OR AB remote consultation OR AB remote consultations OR AB teleconsult OR AB teleconsultation OR AB teleconsultations OR AB tele-consult OR AB tele-consultation OR AB tele-consultations OR AB telemonitoring OR AB tele-monitoring OR AB electronic communication OR AB electronic communications OR AB electronic mail OR AB email OR AB e-mail OR AB emailing OR AB e-mailing OR AB telecommunication OR AB telecommunications OR AB tele-communication OR AB tele-communications OR AB teleconference OR AB teleconferences OR AB teleconferencing OR AB tele-conference OR AB tele-conferences OR AB tele-conferencing OR AB videoconference OR AB videoconferences OR AB videoconferencing OR AB video-conference OR AB video-conferences

Embase

'health care planning'/exp OR 'program evaluation'/exp OR 'nursing evaluation research'/exp OR 'health care policy'/exp OR 'health care delivery'/exp OR 'information dissemination'/exp OR 'translational research'/exp OR implement:ab,ti OR implementation:ab,ti OR implemented:ab,ti OR implementing:ab,ti OR diffuse:ab,ti OR diffused:ab,ti OR diffusion:ab,ti OR disseminate:ab,ti OR disseminated:ab,ti OR disseminating:ab,ti OR dissemination:ab,ti OR upscale:ab,ti OR up-scale:ab,ti OR uptake:ab,ti OR up-take:ab,ti OR translation:ab,ti OR translational:ab,ti OR (program NEAR/2 evaluation):ab,ti OR (process NEAR/2 evaluation):ab,ti OR normalisation:ab,ti OR normalise:ab,ti OR normalised:ab,ti OR normalising:ab,ti OR normalization:ab,ti OR normalize:ab,ti OR normalized:ab,ti OR normalizing:ab,ti OR mainstream:ab,ti OR mainstreamed:ab,ti OR mainstreaming:ab,ti OR maintainance:ab,ti OR maintained:ab,ti OR sustain:ab,ti OR sustainability:ab,ti OR sustainable:ab,ti OR sustained:ab,ti OR integrate:ab,ti OR integrated:ab,ti OR integrating:ab,ti OR integration:ab,ti OR (nursing NEAR/2 evaluation NEAR/2 research):ab,ti OR (health NEAR/1 care NEAR/2 reform):ab,ti OR (delivery NEAR/2 health NEAR/3 care):ab,ti OR (delivery NEAR/2 healthcare):ab,ti OR (organizational NEAR/2 innovation):ab,ti OR (health NEAR/1 care NEAR/2 planning):ab,ti OR (diffusion NEAR/2 innovation):ab,ti OR (health NEAR/1 care NEAR/2 policy):ab,ti

AND

'mental health service'/exp OR 'hospital patient'/exp OR 'outpatient'/exp OR 'social psychiatry'/exp OR 'hospitalization'/exp OR (mental NEAR/1 health NEAR/2 service):ab,ti OR (mental NEAR/1 health NEAR/2 services):ab,ti OR (mental NEAR/1 healthcare):ab,ti OR (mental NEAR/1 health NEAR/1 care):ab,ti OR (mental NEAR/2 practice):ab,ti OR (mental NEAR/2 practices):ab,ti OR (mental NEAR/2 service NEAR/2 delivery):ab,ti OR (inpatient NEAR/1 care):ab,ti OR (in-patient NEAR/1 care):ab,ti OR (out-patient NEAR/1 care):ab,ti OR (outpatient NEAR/1 care):ab,ti OR (mental NEAR/1 health NEAR/2 program):ab,ti OR (mental NEAR/1 health NEAR/2 programme):ab,ti OR (mental NEAR/1 health NEAR/2 programmes):ab,ti OR (mental NEAR/1 health NEAR/2 programs):ab,ti OR (community NEAR/2 psychiatry):ab,ti OR (community NEAR/2 psychology):ab,ti OR (community NEAR/1 based):ab,ti OR (hospital NEAR/1 patient):ab,ti OR (social NEAR/2 psychiatry):ab,ti OR 'mood disorder'/exp OR (mood NEAR/2 disorder):ab,ti OR (mood NEAR/2 disorders):ab,ti OR depressed:ab,ti OR depressing:ab,ti OR depression:ab,ti OR depressions:ab,ti OR depressive:ab,ti OR bipolar:ab,ti OR mania:ab,ti OR manic:ab,ti

AND

'telecommunication'/exp OR 'computer system'/exp OR 'mobile application'/exp OR telehealth:ab,ti OR tele-health:ab,ti OR telemedicine:ab,ti OR tele-medicine:ab,ti OR e-health:ab,ti OR ehealth:ab,ti OR emental:ab,ti OR e-mental:ab,ti OR mhealth:ab,ti OR m-health:ab,ti OR (web-based NEAR/2 intervention):ab,ti OR (web-based NEAR/2 interventions):ab,ti OR (web-based NEAR/2 treatment):ab,ti OR (web-based NEAR/2 treatments):ab,ti OR (web-based NEAR/2 therapy):ab,ti OR (web-based NEAR/2 therapies):ab,ti OR (internet NEAR/2 intervention):ab,ti OR (internet NEAR/2 interventions):ab,ti OR (internet NEAR/2 treatment):ab,ti OR (internet NEAR/2 treatments):ab,ti OR (internet NEAR/2 therapy):ab,ti OR (internet NEAR/2 therapies):ab,ti OR (internet-based NEAR/2 intervention):ab,ti OR (internet-based NEAR/2 interventions):ab,ti OR (internet-based NEAR/2 treatment):ab,ti OR (internet-based NEAR/2 treatments):ab,ti OR (internet-based NEAR/2 therapy):ab,ti OR (internet-based NEAR/2 therapies):ab,ti OR (online NEAR/2 intervention):ab,ti OR (online NEAR/2 interventions):ab,ti OR (online NEAR/2 treatment):ab,ti OR (online NEAR/2 treatments):ab,ti OR (online NEAR/2 therapy):ab,ti OR (online NEAR/2 therapies):ab,ti OR (computer-based NEAR/2 intervention):ab,ti OR (computer-based NEAR/2 interventions):ab,ti OR (computer-based NEAR/2 treatment):ab,ti OR (computer-based NEAR/2 treatments):ab,ti OR (computer-based NEAR/2 therapy):ab,ti OR (phone-based NEAR/2 intervention):ab,ti OR (phone-based NEAR/2 interventions):ab,ti OR (phone-based NEAR/2 treatment):ab,ti OR (telephone-based NEAR/2 intervention):ab,ti OR (telephone-based NEAR/2 interventions):ab,ti OR (telephone-based NEAR/2 treatment):ab,ti OR (smartphone-based NEAR/2 intervention):ab,ti OR (smartphone-based NEAR/2 interventions):ab,ti OR guided:ab,ti OR unguided:ab,ti OR blended:ab,ti OR (computer NEAR/2 mediated):ab,ti OR (computer NEAR/2 assisted):ab,ti OR (computer NEAR/2 augmented):ab,ti OR econsult:ab,ti OR econsultation:ab,ti OR econsultations:ab,ti OR e-consult:ab,ti OR e-consultation:ab,ti OR e-consultations:ab,ti OR (remote NEAR/2 consultation):ab,ti OR (remote NEAR/2 consultations):ab,ti OR teleconsult:ab,ti OR teleconsultation:ab,ti OR teleconsultations:ab,ti OR tele-consult:ab,ti OR tele-consultation:ab,ti OR tele-consultations:ab,ti OR telemonitoring:ab,ti OR tele-monitoring:ab,ti OR (electronic NEAR/2 communication):ab,ti OR (electronic NEAR/2 communications):ab,ti OR (electronic NEAR/2 mail):ab,ti OR email:ab,ti OR e-mail:ab,ti OR emailing:ab,ti OR e-mailing:ab,ti OR telecommunication:ab,ti OR telecommunications:ab,ti OR tele-communication:ab,ti OR tele-communications:ab,ti OR teleconference:ab,ti OR teleconferences:ab,ti OR teleconferencing:ab,ti OR tele-conference:ab,ti OR tele-conferences:ab,ti OR tele-conferencing:ab,ti OR videoconference:ab,ti OR

videoconferences:ab,ti OR videoconferencing:ab,ti OR video-conference:ab,ti OR video-conferences:ab,ti

References

1. Greenhalgh, T., Robert, G., Macfarlane, F., Bate, P., & Kyriakidou, O. (2004, January 1). Diffusion of innovations in service organizations: systematic review and recommendations. The Milbank Quarterly. doi:10.1111/j.0887-378X.2004.00325.x

2. May, C., & Finch, T. (2009, June 15). Implementing, Embedding, and Integrating Practices: An Outline of Normalization Process Theory. Sociology. doi:10.1177/0038038509103208

3. Rogers, E. M. (2003, August 16). Diffusion of Innovations, 5th Edition. Retrieved from http://books.google.nl/books?id=9U1K5LjUOwEC&printsec=frontcover&dq=inauthor:Rogers+intitle:Diffusion+of+innovations&hl=&cd=1&source=gbs_api

4. Damanpour, F., & Gopalakrishnan, S. (1998, March 1). Theories of organizational structure and innovation adoption: the role of environmental change. Journal of Engineering and Technology Management. doi:10.1016/S0923-4748(97)00029-5

5. Meyer, A. D., & Goes, J. B. (1988, December 1). Organizational Assimilation of Innovations: A Multilevel Contextual Analysis. The Academy of Management Journal. doi:10.2307/256344?ref=no-x-route:d209b01f4069eb5c95cda93e2cd4d484

6. Greenhalgh, T., Robert, G., Bate, P., Macfarlane, F., & Kyriakidou, O. (2005, January 1). Diffusion of Innovations in Health Service Organisations. Retrieved from http://books.google.nl/books?id=poNvGuSLlOkC&dq=isbn:0727918699&hl=&cd=1&source=gbs_api

7. Riper, H., Christensen, H., Cuijpers, P., Lange, A., & Eysenbach, G. (2010, January 1). Theme Issue on E-Mental Health: A Growing Field in Internet Research. Journal of Medical Internet Research. doi:10.2196/jmir.1713

8. Glasgow, R. E., Vogt, T. M., & Boles, S. M. (1999, September 1). Evaluating the public health impact of health promotion interventions: The RE-AIM framework. American Journal of Public Health. doi:10.2105/AJPH.89.9.1322

9. Belza, B., Toobert, D., & Glasgow, R. E. (2014, October 28). RE-AIM for Program Planning: Overview and Applications.

10. Gaglio, B., Shoup, J. A., & Glasgow, R. E. (2013, June 1). The RE-AIM Framework: A Systematic Review of Use Over Time. American Journal of Public Health. doi:10.2105/AJPH.2013.301299

11. Jilcott, S., Ammerman, A., Sommers, J., & Glasgow, R. E. (2007, October 1). Applying the RE-AIM framework to assess the public health impact of policy change. Annals of Behavioral Medicine : A Publication of the Society of Behavioral Medicine. doi:10.1080/08836610701564055
